# Neuroinflammation in Alzheimer's Disease: Mechanisms, Impact and Emerging Therapies

**DOI:** 10.1002/alz70855_105403

**Published:** 2025-12-24

**Authors:** Aryan Malik

**Affiliations:** ^1^ MMCP, Ambala, Haryana, India

## Abstract

**Background:**

Alzheimer's disease (AD) is a prevalent neurodegenerative disorder and a serious form of dementia, posing a significant threat to global health due to its unknown etiology.

**Methods:**

This review examines the molecular mechanisms underlying AD progression, including the RIPK/MLKL axis, autophagy, necroptosis, and neuroinflammation.

**Results:**

Uncontrolled neuronal cell death triggers dysregulated cellular activity and synaptic dysfunction, contributing to AD progression. The RIPK/MLKL axis, autophagy, and necroptosis play critical roles in this process. Neuroinflammation, mediated by pro‐inflammatory cytokine signaling, accelerates AD pathogenesis through signaling pathways such as NFκB and p38 MAPK.

**Conclusion:**

Understanding the complex interplay between these molecular mechanisms is crucial for developing effective therapeutic strategies for AD treatment. Current pharmacological treatments focus on symptom alleviation, and novel approaches targeting disease modification are urgently needed.

Here are the keywords in alphabetical order:

**Keywords**: Dementia, Memory impairments, neuropsychiatric disorders, p38 MAPK, pro‐apoptotic caspases, pro‐inflammatory mediators.

